# Commentary: The role of single nucleotide polymorphisms related to iron homeostasis in mesothelioma susceptibility after asbestos exposure: a genetic study on autoptic samples

**DOI:** 10.3389/fpubh.2024.1336545

**Published:** 2024-01-16

**Authors:** Georgios Tagarakis, Fani Tsolaki, Ioannis Tagarakis

**Affiliations:** ^1^Department of Cardiothoracic Surgery, Aristotle University of Thessaloniki, Thessaloniki, Greece; ^2^“Health and Social Care Services Management”, Aristotle University of Thessaloniki, Thessaloniki, Greece; ^3^Democritus University of Thrace, Alexandroupolis, Greece

**Keywords:** asbestos, mesothelioma, iron metabolism, malignancy, environment

## Introduction

Malignant pleural mesothelioma remains one of the most scientifically interesting and controversial diseases in regard to pathogenesis, prevention and treatment. The role of iron metabolism in the development of this malignancy after asbestos exposure has been supported by various authors. However, recent findings debate the connection between iron and the disease. The scope of this commentary is to express our opinion on the matter taking as a starting point the interesting article by Grignani et al. (1).

## Relationship between iron, asbestos and mesothelioma

The recent article by Grignani et al. (1) deals with the role of iron- related genetic polymorphisms after asbestos exposure in the genesis of malignant pleural mesothelioma. Some interesting related issues we would like to comment on are the following:

(i) Malignant pleural mesothelioma remains one of the most controversial and scientifically interesting malignancies as far as pathogenesis, prevention and treatment are concerned. Well-known are, for example, the long debates about the role of extended surgical therapy in form of extrapleural pneumonectomy in comparison with less radical procedures (pleurectomy/decortication) (2) and the role of the still under investigation novel therapies in form of monoclonal antibodies (3).
(ii) The case of asbestos is one of the most classical paradigms relating environmental factors with a disease (4); the asbestos removal campaign has made a great progress, especially in Europe, however must be further expanded toward the target of total eradication (5).
(iii) The role of iron metabolism in the development of mesothelioma has been supported by various authors; it is believed that iron in combination with asbestos exposure induces morbid pathways related to oxidative stress and ferroptosis (6). In this context, the article (1) deals with a hot scientific topic interesting for all scientists fighting the disease. The set-up of the commented study, involving active and control groups and the sufficient sample size, despite some limitations (e.g., the localization of the study) render the results trustworthy and let the debate about the involvement of iron metabolism in the development of malignant pleural mesothelioma pending, awaiting further, larger studies to solve it.

## Discussion

Malignant pleural mesothelioma is an aggressive malignancy that has been associated with various controversies in regard to its prevention, diagnosis and treatment (2, 3). Among these controversies one could include the amphibolous role of iron homeostasis after exposure to asbestos in the development of mesothelioma (1, 6). Larger studies, involving more data deriving from a larger sample of patients may elucidate the matter, which could lead to a better preventive or therapeutic approach of malignant pleural mesothelioma.

## Author contributions

GT: Conceptualization, Writing – original draft, Writing – review & editing. FT: Investigation, Writing – review & editing. IT: Investigation, Writing – review & editing.
